# A Novel Synthetic Analog of 5, 8-Disubstituted Quinazolines Blocks Mitosis and Induces Apoptosis of Tumor Cells by Inhibiting Microtubule Polymerization

**DOI:** 10.1371/journal.pone.0010499

**Published:** 2010-05-05

**Authors:** Wei Tian, Lili Qin, Qiaoling Song, Li He, Midan Ai, Yi Jin, Zuyu Zhou, Song You, Yaqiu Long, Qiang Yu

**Affiliations:** 1 Shanghai Institute of Materia Medica, Chinese Academy of Sciences, Shanghai, China; 2 The School of Life Science and Biopharmaceutics of Shenyang Pharmaceutical University, Liaoning, China; National Cancer Institute, United States of America

## Abstract

Many mitosis inhibitors are powerful anticancer drugs. Tremendous efforts have been made to identify new anti-mitosis compounds for developing more effective and less toxic anti-cancer drugs. We have identified LJK-11, a synthetic analog of 5, 8-disubstituted quinazolines, as a novel mitotic blocker. LJK-11 inhibited growth and induced apoptosis of many different types of tumor cells. It prevented mitotic spindle formation and arrested cells at early phase of mitosis. Detailed *in vitro* analysis demonstrated that LJK-11 inhibited microtubule polymerization. In addition, LJK-11 had synergistic effect with another microtubule inhibitor colchicine on blocking mitosis, but not with vinblastine or nocodazole. Therefore, LJK-11 represents a novel anti-microtubule structure. Understanding the function and mechanism of LJK-11 will help us to better understand the action of anti-microtubule agents and to design better anti-cancer drugs.

## Introduction

Microtubules are critical elements in a variety of fundamental cellular functions, including formation and maintenance of cell shape, transportation of vesicles and protein complexes, and regulation of motility and cell division[1]. Microtubules are extremely important in the process of mitosis, during which the duplicated chromosomes of a cell are separated into two identical sets before cleavage of the cell into two daughter cells. The essential role of microtubules in mitosis and cell division makes them and their regulatory proteins important, perhaps the best, targets for anticancer drugs.Anti-microtubule agents, such as nocodazole, vinorelbine, colchicines, and paclitaxel, bind microtubules, deregulate microtubule dynamics, activate mitotic spindle checkpoint, and induce cell apoptosis[2]–[4]. Many studies have confirmed that suppression of microtubule dynamics and interference of microtubule polymerization or depolymerization seems to be an effective why to block mitosis and kill tumor cells[5], [6]. The anti-microtubule agents have been successfully used in treating a variety of cancers[7], [8].

Microtubule-targeted anti-mitotic drugs are usually classified into two main groups based on their mode of action. One group, known as microtubule-destabilizing agents, inhibits microtubule polymerization and promotes microtubule depolymerization, such as vinca alkaloids. The second group, represented by taxanes and characterized as microtubule-stabilizing agents, inhibits microtubule depolymerization and stabilizes microtubules. They are also classified based on their binding sites on tubulin[8]–[10]. The anti-microtubule agents affect microtubule-polymer mass as well as their dynamics. The effects on microtubule dynamics are often more powerful than the effects on polymer mass in treating cancer cells[11]–[13].

Although all of the anti-microtubule agents inhibit microtubule dynamics in vitro effectively, their effects against different types of cancers in vivo vary [14]. In addition, despite the success of taxanes and vinca alkaloids to inhibit the progression of some cancers in clinical use, resistance to anti-microtubule agents is encountered in many tumor types, particularly during multiple cycles of therapy[15]–[18]. Therefore, there has been great interest in identifying and developing novel anti-microtubule drugs.

We have screened for growth inhibitory compounds and identified LJK-11, an analog of 5, 8-disubstituted quinazolines, as a cell mitosis blocker. Analysis of the function and mechanism of LJK-11 revealed that LJK-11 is a microtubule- destabilizing agent. It inhibits microtubule polymerization, arrests cells at early stage of mitosis, and induces apoptosis. Our data suggest that LJK-11 is a novel anti-microtubule compound. Understanding the mechanism of the LJK-11 will increase our knowledge about the anti-microtubule agents and will help us to design better anti-cancer drugs.

## Results

### LJK-11 inhibited growth and induced apoptosis of human tumor cells

**Figure 1:**
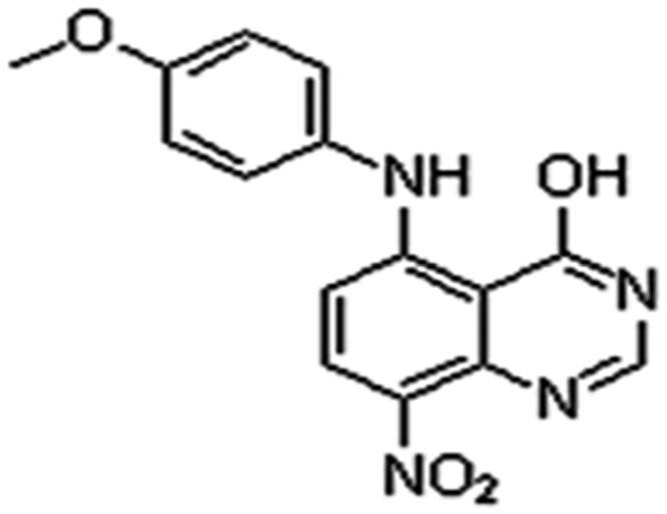
Chemical structure of LJK-11.

LJK-11 was synthesized as an analog of 5, 8-disubstituted quinazolines (Fig. 1). The chemical formula of LJK-11 is C_15_H_12_N_4_O_4_ yielding a molecular weight of 312.28. Quinazolines are a class of fused heterocycles which display diverse range of biological activities. Among them, 4-anilinoquinazolines are the most promising small molecule EGFR tyrosine kinase inhibitors. The 5-substituted quinazoline derivatives, however, have no effect on the EGFR tyrosine kinase, but display potent antiproliferative effect[19], [20] .

**Figure 2:**
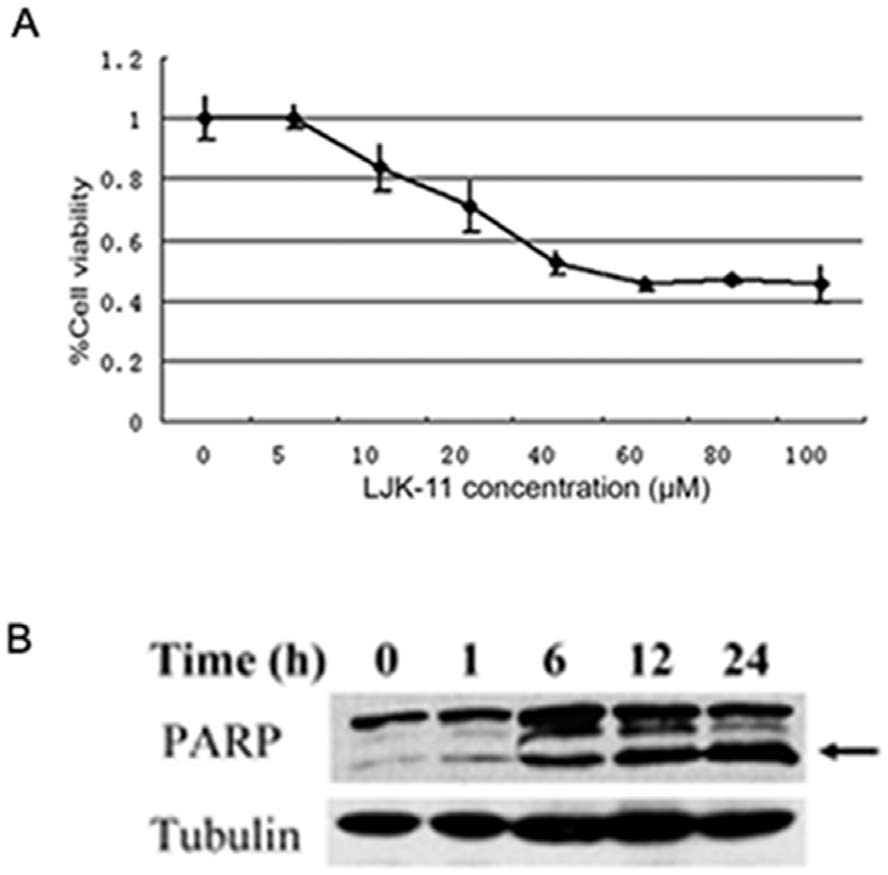
Effect of LJK-11 on the growth and death of A549 cells. A. MTT assay of LJK-11 effects on cell growth. A549 cells were incubated with indicated concentrations of LJK-11 for 48 hours. The effect on cell growth and death was examined by MTT assay. The cell viability is expressed as a percentage of the compound-treated viable cells divided by the viable cells of the untreated control. The data are the means of triplicates ±SD. B. PARP cleavage assay of LJK-11 effects on inducing cell apoptosis. A549 cells were treated with 50 µM for indicated time periods. The cell lysates were resolved by SDS-PAGE and analyzed by Western bolt analysis using anti-PARP antibody. The cleaved PARP is indicated by the arrow. Anti-tubulin antibody was used as a protein loading control.

To understand the mechanism of the 5-substituted quinazoline derivatives in inhibiting cell growth, we analyzed the effect of the 5-substituted quinazoline derivatives on the growth of a human lung adenocarcinoma cell line A549 and identified LJK-11 as the most effective compound. The growth of the A549 cells was inhibited by LJK-11 in a dose-dependent manner with an IC_50_ of about 20 µM (Fig. 2A). Further analysis demonstrated that LJK-11 also induced apoptosis in A549 cells (Fig. 2B). The apoptosis, as indicated by the cleavage of PARP, occurred 24 hours after LJK-11 treatment (Fig. 2B).

**Figure 3:**
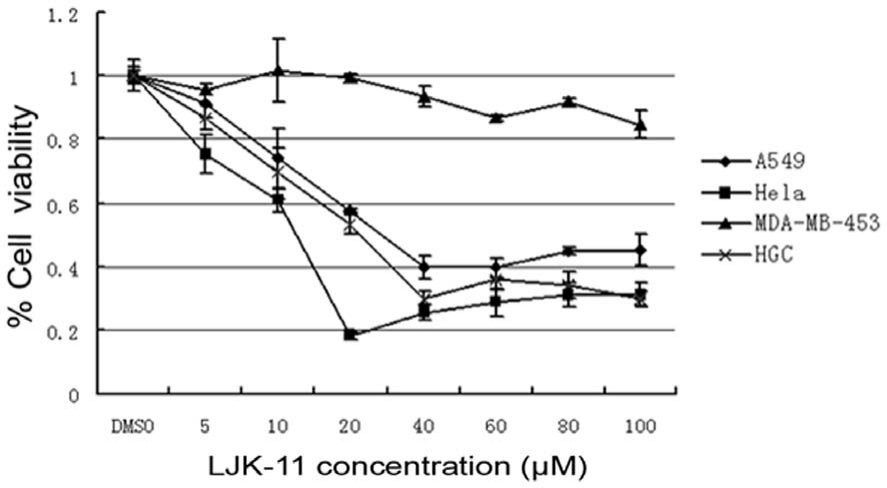
Effect of LJK-11 on the growth and death of different tumor cells. A549, Hela, HGC-27, or MDA-MB-453 cells were incubated with the indicated concentrations of LJK-11 for 48 hours. The effect of LJK-11 on cell growth and death was evaluated by MTT assay. The cell viability is expressed as a percentage of the compound-treated viable cells divided by the viable cells of the untreated control. The data are the means of triplicates ±SD.

We then analyzed the cell specificity of LJK-11 using human tumor cell lines of different tissue origins, including the lung adenocarcinoma cell line A549, cervical cancer cell line Hela, gastrointestinal cancer cell line HGC-27, and breast cancer cell line MDA-MB-453. Our data indicated that LJK-11 inhibited the growth of A549, Hela, and HGC-27 equally, while had less effect on MDA-MB-453 cells (Fig. 3). These results demonstrated that LJK-11 inhibits growth of most of the tumor cells.

**Figure 4:**
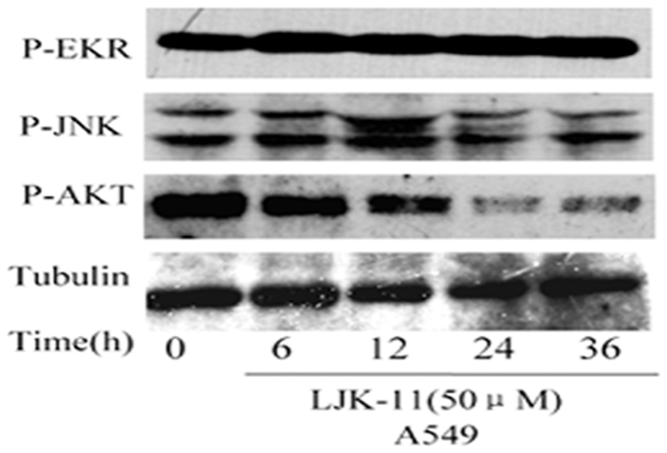
Effect of LJK-11 on tyrosine phosphorylation of signaling proteins. A549 cells were treated with 50 µM LJK-11 for 6, 12, 24, or 36 hours. The cell lysates were resolved by SDS-PAGE and analyzed by Western bolt analysis using antibodies as indicated. Antibodies specific to the phosphorylated forms of the indicated proteins are labeled with (-P).

To explore the mechanism of the growth inhibition and cell death effects of LJK-11 on tumor cells, we analyzed the expression and phosphorylation of several major cell growth and survival signaling proteins along the tyrosine kinase signaling pathway, including ERK, JNK, and AKT. The results indicated that LJK-11 had no obvious effect on the phosphorylation of JNK, but increased the phosphorylation of ERK 1/2 and decreased the phosphorylation of AKT (Fig. 4), suggesting that inhibition of the PI3K/AKT pathway may be one of the mechanisms that responsible for the LJK-11-induced growth arrest and/or cell death.

### LJK-11 arrested cells at G2/M phase

**Figure 5:**
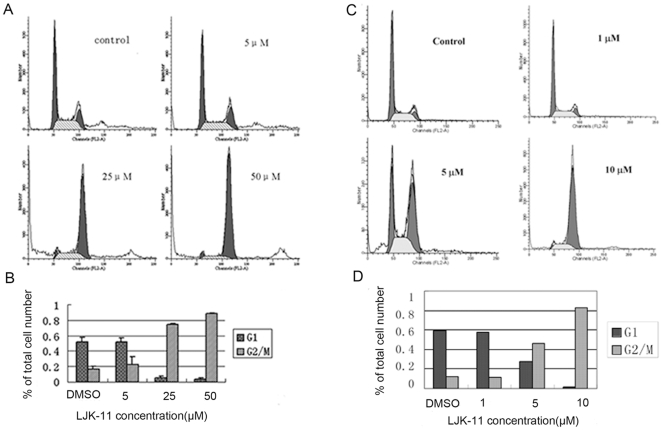
Effects of LJK-11 on cell cycle distribution. A. Flow cytometry analysis of LJK-11-treated A549 tumor cells. A549 cells were incubated with different concentrations of LJK-11 for 24 hours. The cells were then fixed and stained with PI, and analyzed by flow cytometry. B. Percentage of cells in G2/M phase after 24 hours treatment with different concentrations of LJK-11. The data are the means of triplicates ±SD. C. Flow cytometry analysis of LJK-11-treated MDA-MB-453 tumor cells. MDA-MB-453 cells were incubated with different concentrations of LJK-11 for 24 hours. The cells were then fixed and stained with PI, and analyzed by flow cytometry. D. Percentage of cells in G2/M phase after 24 hours treatment with different concentrations of LJK-11. The data are the representative of three independent experiments.

We observed gradual accumulation of rounded cells, which resemble the appearance of mitotic cells, after addition of LJK-11. We therefore analyzed the effect of LJK-11 on cell cycle[21]. LJK-11 induced a dose-dependent G2/M arrest after 24 hours of drug exposure. 88% of the cell population was blocked in G2/M phase when they were exposed to 50 µM LJK-11 for 24 hours (Fig. 5A and B).

To test whether the anti-mitotic activity of LJK-11was universal to all tumor cells, we further analyzed the effect of LJK-11 on the cell cycle progression of the MDA-MB-453 cells, which was relatively insensitive to LJK-11 treatment in the MTT assay. (Fig. 3). As shown in Figure 5, LJK-11 also effectively blocked the MDA-MB-453 cells at G2/M phase (Fig. 5C and D). These data clearly indicate that LJK is a mitotic blocker.

### LJK-11 disrupted mitotic spindles in cells and inhibited tubulin polymerization *in vitro*

**Figure 6:**
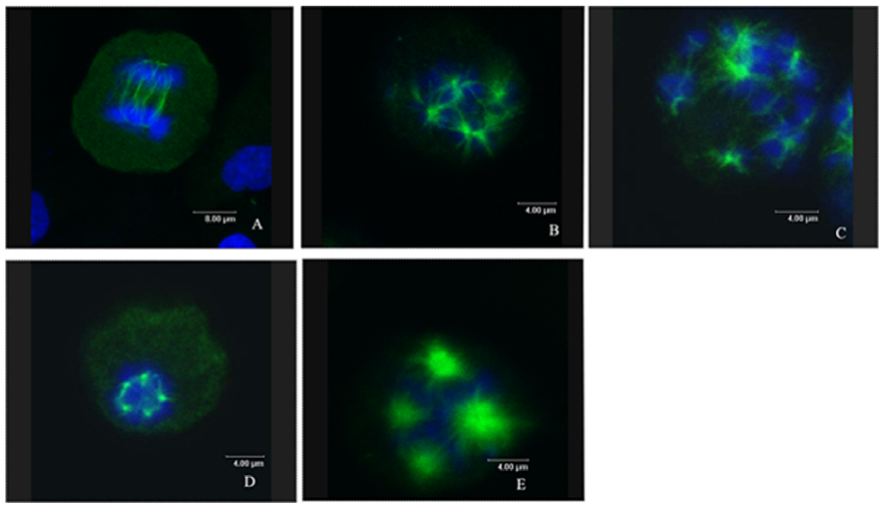
Effects of LJK-11 on mitotic spindle formation. A549 cells were incubated on glass coverslips with different reagents for 16 hours, and then fixed and stained with α-tubulin antibody to visualize microtubules (green) and with DAPI to visualize chromosomes (blue). The cells were visualized by indirect immunofluorescent microscopy. A: control cells treated with equal volume of DMSO (0.1%). B: cells treated with 100 µM LJK-11. C: cells treated with 5 nM nocodazole. D: cells treated with 100 nM colchicine. E: cells treated with 50 nM Taxol.

**Figure 7:**
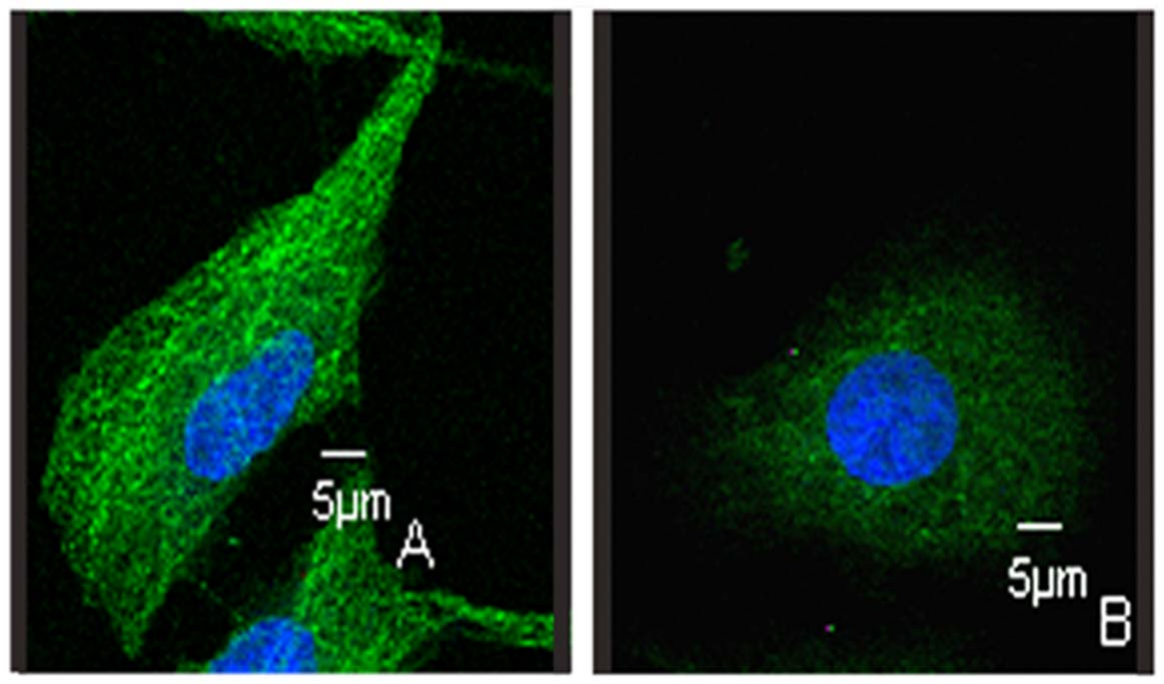
Effects of LJK-11 on tubulin structure in non-mitotic cells. A549 cells were incubated on glass coverslips with (B) or without (A) LJK-11 for 4 hours, and then fixed and stained with α-tubulin antibody to visualize microtubules (green) and with DAPI to visualize chromosomes (blue). The cells were visualized by indirect immunofluorescent microscopy.

Most of the anti-mitotic agents interact with microtubules[22]. The effect of LJK-11 on microtubule structure was then examined by immuno-fluorescence microscopy using an α-tubulin antibody. Normal control cells in metaphase displayed a bipolar mitotic spindle. Condensed chromosomes lined up between the spindle poles to form the metaphase plate (Fig. 6A). Cells exposed to LJK-11 for 16 hours displayed disrupted appearance of mitotic spindles (Fig. 6B). To compare the effect of LJK-11 with other mitotic blockers, cells were treated with different concentrations of nocodazole, taxol or colchicine for 16 hours. Taxol, a microtubule-stabilizing agent, increased microtubules and induced multi-polar spindles (Fig. 6E)[23]. Colchicine and nocodazole, two microtubule depolymerizing agents[24], [25], caused multipolar mitotic spindle formation (Fig. 6C and D). LJK-11 (Fig. 6B) also caused multipolar mitotic spindle formation, similar to that of colchicines and nocodazole, suggesting that LJK-11 is a microtubule depolymerizing agent. Further more, the microtubule structure in interphase cells were also disrupted (Fig. 7), further confirming that LJK-11 interacted with tubulin directly.

**Figure 8:**
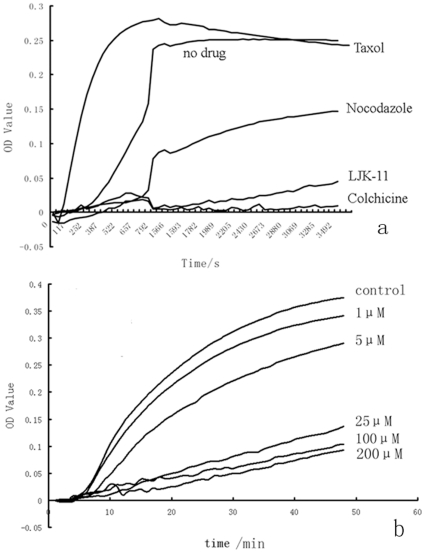
Effect of LJK-11 on tubulin polymerization. Effects of LJK-11 (250 µM), colchicines (10 µM), nocodazole (10 µM), or Taxol (10 µM) on bovine brain tubulin polymerization were measured turbidimetrically(A). Effects of 1 µM, 5 µM, 25 µM, 100 µM, 200 µM LJK-11 on bovine brain tubulin polymerization were also measured. Changes in absorbance at 340 nm (A_340_) were measured and plotted as a function of time(B).

To confirm the above observation, we investigated the effect of LJK-11 on tubulin polymerization using an in vitro tubulin polymerization assay (Fig. 8). LJK-11 inhibited polymerization of microtubules in a dose-dependent manner. Taken together, our data indicate that LJK-11 is a microtubule assembly inhibitor.

**Figure 9:**
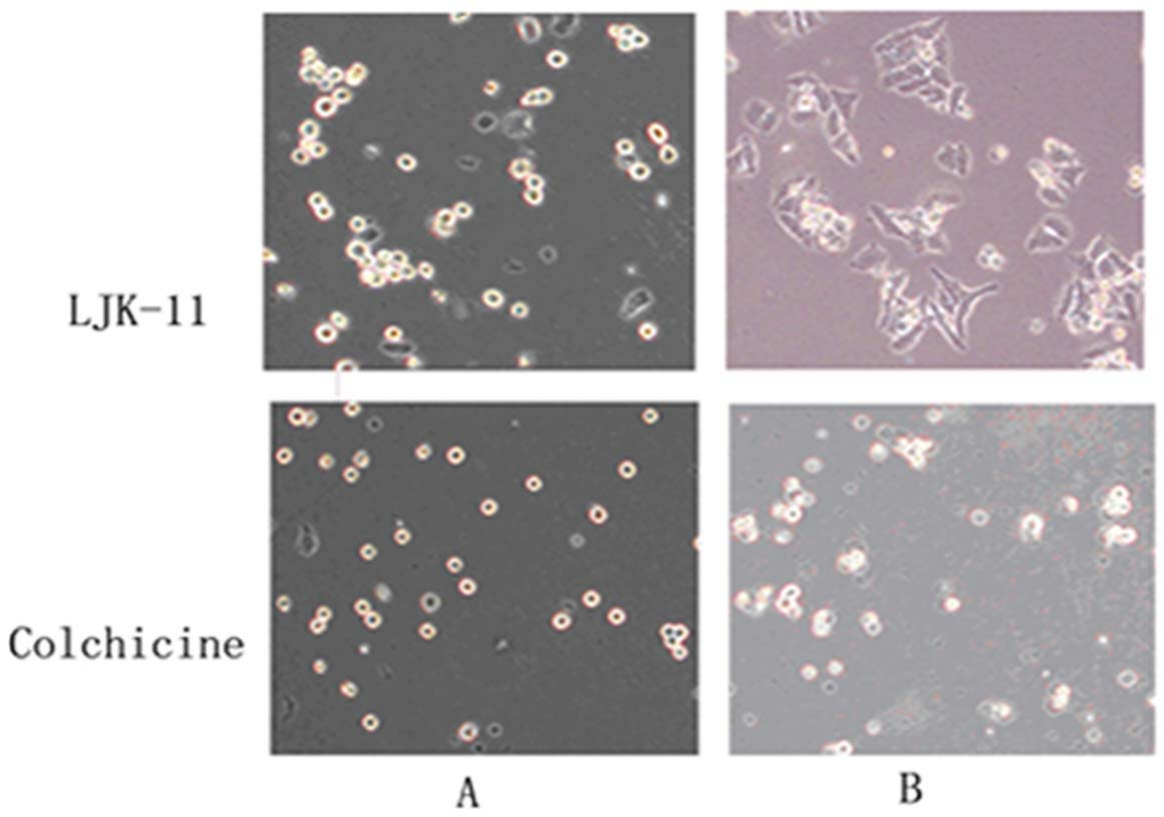
Reversibility of LJK-11 treatment. Hela cells were incubated with 50 µM LJK-11 or 20 nM colchicine for 20 hours (A) and then the compound-containing media were removed, the cells were washed with fresh media, and the cells were incubated in new compound-free media for additional 18 hours (B). The pictures were taken using a light microscope.

To test whether LJK-11 is a reversible inhibitor, we incubated the cells with LJK-11 for 20 hours and then washed out the LJK-11 and incubated the cells for additional 18 hours. The LJK-11-treated cells grew again after washing, suggesting that the LJK-11 treatment is reversible. One the contrary, the colchicines-treated cells did not recover after washing (Fig. 9)

### LJK-11 and colchicine acted synergistically to induce G2/M arrest

**Figure 10:**
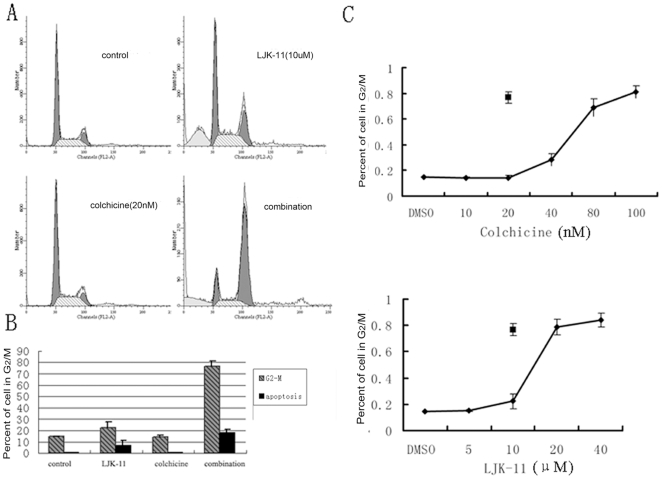
Synergistic effect of LJK-11 and colchicine on blocking mitosis. **A**. Flow cytometry analysis of the effects of LJK-11 (10 µM), colchicines (20 nM), or the combination of the two on cell cycle distribution. A549 cells were incubated with 10 µM LJK-11, 20 nM colchicine, or combination of 10 µM LJK-11 and 20 nM colchicine for 24 hours. The cells were then fixed and stained with PI, and analyzed by flow cytometry. **B**. Percentage of cells in G2/M phase after 24 hours treatment with 10 µM LJK-11, 20 nM colchicine, or combination of 10 µM LJK-11 and 20 nM colchicine. **C**. Concentration dependent G2/M arrest by treatment of colchicines or LJK-11 for 24 h. Also indicated in the figures are the percentages of G2/M arrest induced by the combination of 10 µM LJK-11 and 20 nM colchicine. Data are the means of triplicates ± SD.

To find out whether LJK-11 functions similarly to other known anti-microtubule agents, we analyzed the effects of LJK-11 in combination with other anti-microtubule agents on tumor cell growth and death. We found that combination of LJK-11 and colchicine synergistically increased the percentage of G2/M-arrested cells (Fig. 10).The percentage of cells in G2/M after incubation with the combination of the two drugs (76.8±4.3%) was much greater than that of LJK-11 alone (22.3±5.5%) or colchicine alone (14.2±1.9%) at the same concentration. The Combination Index value (CI = 0.63±0.08, 95% confidence interval) indicated that the combination of LJK-11 and colchicine acted synergistically to block mitosis. On the contrary, combination of LJK-11 with two other microtubule depolimerizing agents, nocodazole or vinblastine sulfate, had neither synergistic nor additive effects (data not shown).

## Discussion

LJK-11 is a novel compound and its structure is different from that of colchicine, nocodazole, vinblastine, or taxol[26], [27]. It was identified from screening a series of growth inhibitory compounds. It inhibits proliferation and induces apoptosis of different types of tumor cell lines. We presented several lines of evidences to demonstrate that LJK-11 blocked mitosis of tumor cells by inhibiting microtubule polymerization.

The exact mechanism by which LJK-11 inhibits microtubule polymerization is not completely understood. The in vitro analysis of the effect of LJK-11 on microtubule polymerization suggests that LJK-11 may interact directly with tubulin. Both microtubule-stabilizing and microtubule–destabilizing agents cause formation of abnormal mitotic spindle and inhibited mitotic progression[25], [28]. The effect of LJK-11 likely indicates inhibition of normal microtubule dynamics and microtubule polymerization [29], [30]. Visualization of mitotic spindle with fluorescent microscopy indicated that LJK-11 caused multipolar mitotic spindles. The mode of action on inhibiting microtubule polymerization and the effect on mitotic spindle formation of LJK-11 resemble most that of colchicines[31]. However, the fact that LJK-11 acted synergistically with colchicine on blocking mitosis suggests that LJK-11 may interact with a different site of tubulin from that of colchicines[32]–[34]. Taken together, these data suggest that LJK-11 is a new type of microtubule depolymerizing agent and is different from colchicine.

In addition to its anti-mitosis activity, LJK-11 also induced apoptosis of most of the tumor cells. To understand better the mechanism of the LJK-11-induced apoptosis, we also analyzed the effects of LJK-11 on phosphorylation/activation of several cell growth and survival-related signaling proteins, including ERK1/2., JNK, and AKT. LJK-11 had no obvious effect on the phosphorylation of JNK, but increased the phosphorylation of ERK1/2 and decreased the phosphorylation of AKT. The effects of LJK-11 on JNK and ERK1/2 are also different from that of the mitotic blockers colchicine, vinblastine, or taxol, all of which increased phosphorylation of JNK while decreased phosphorylation of ERK1/2[35], [36]. Although it is not clear what role the ERK activation may play in the LJK-11-induced apoptosis, our data suggest that LJK-11 has different effects on cellular signaling system in comparison with other mitotic blockers and that inhibition of AKT phosphorylation/activation may be the key for LJK-11 to induce apoptosis of tumor cells. Under this notion, it is interesting to note that the apoptosis-inducing activity of LJK-11 appeared to be selective comparing to its universal anti-mitosis activity. Some of the tumor cells, such as MDA-MB-453, were resistant to the LJK-11-induced cell death. Further investigation into the correlation between the AKT activation and the sensitivity to the LJK-11-induced apoptosis in various tumor cells will help to evaluate its potential as an anti-cancer drug.

In summary, we have identified a new type of microtubule depolymerizer and a mitotic blocker. Understanding of the mechanism of LJK-11 on inhibiting microtubule polymerization and induction of apoptosis of tumor cells and its differences from other microtubule polymerization inhibitors will help us to design better anti-cancer drugs.

## Materials and Methods

### Chemical synthesis of compound LJK-11

LJK-11 (MW: 312.28) was synthesized via a straightforward six-step synthetic route as reported previously[19], [20], starting from the commercially available 3-chloro-2-methyl aniline and employing Neimentowski synthesis as the key step. The final product was characterized by HNMR, MS, and Elemental analyses. All other chemicals were purchased from Sigma Chemical Co.

### Cell culture and reagents

Human lung adenocarcinoma cell line A549, cervical cancer cell line Hela, and breast cancer cell line MDA-MB-453 were purchased from ATCC; gastrointestinal cell line HGC-27 was provided by the Fourth Military Medical University of China. A549 cells were cultured in RPMI 1640 medium supplemented with 10% FBS; other cell lines were cultured in DMEM medium supplemented with 10% FBS.

### MTT assay

Cells were seeded in a 96-well plate (5×10^3^ cells/well) and cultured overnight, then treated with various concentrations of LJK-11 and incubated for additional 48 hours. The MTT (3-(4, 5-dimethylthiazol–2-yl) -2, 5-diphenyl tetrazolium bromide) assay was performed by adding 20 µl of MTT solution (5 mg/ml, Sigma Chemical Co.) to each well and incubated for 3 hours at 37°C. The supernatant was aspirated, and the MTT-formazan crystals formed by metabolically viable cells were dissolved in 150 µl of DMSO. The absorbance was measured by a microplate reader at a wavelength of 570 nm.

### Flow Cytometry analysis

The cells were harvested and washed with PBS, resuspended in 1 ml of ice-cold 75% ethanol. After being left to stand overnight, cell pellets were collected by centrifugation, resuspended in 500 µl of hypotonic buffer (0.5% Triton X-100 in PBS and 0.5 µg/ml RNase), and incubated at 37°C for 30 min. Then 25 µl of propidium iodide solution (50 µg/ml) was added, and the mixture was allowed to stand on ice for 1 hour. Fluorescence emitted from the propidium iodide-DNA complex was quantitated after excitation of the fluorescent dye by FAC-Scan cytometry. The histogram of DNA distribution was modeled as a sum of G1, G2/M, S phase, and an sub-G1 population, by using ModFitLT software.

### Immunofluorescence microscopy

After culturing for 48 hours on cover-slips, A549 cells were incubated with drugs at various concentrations for 16 hours. Cells were then fixed. After blocking, cells were incubated with mouse monoclonal α-tubulin antibody for 2 hours at 37°C. The secondary antibody, Fluorescein (FITC)-conjugated affinipure goat anti-mouse IgG (H+L), was added and incubated for 1 hour. Chromosomes were stained with 1 µg/ml DAPI in PBS. After washing with PBS, the slides were mounted and sealed. Fluorescence images were captured by using Leica TCS SP2 laser confocal microscope.

### Western Blot Analysis

Cells were lysed in the ice-cold cell lysis buffer (PH 7.6) containing 0.5 mM dithiothreitol, 0.2 mM EDTA, 20 mM HEPES, 2.5 mM MgCl_2_,75 mM NaCl, 0.1 mM Na_3_VO_4_, 50 mM NaF, and 0.1% Triton X-100. The protease inhibitors including 1 µg/ml aprotinin, 0.5 µg/ml leupeptin, and 100 µg/ml 4-(2-aminoethyl)-benzenesulfonyl fluoride were added to the cell suspension. The cell extracts were gently rotated at 4°C for 30 min. After centrifugation, the pellets were discarded. Equal amounts of proteins were subjected to 8–10% SDS-PAGE. After transfer of proteins onto nitrocellulose membranes, they were hybridized with various antibodies according to the instructions provided by the manufacturers.

### *In vitro* tubulin polymerization assay

The assay was essentially performed according to Ching-Chuan Kuo et al [37]. Briefly, the sample (100 µl of 3 mg/ml tubulin proteins) in TP buffer (100 mM PIPES, pH 6.9, 2 mM MgCl2, 1 mM GTP, and 15% glycerol) was placed in 96-well microtiter plates in the presence of test agents. Mixtures were warmed to 37°C and the increase in absorbance was measured at 340 nm in TECAN Genois Pro Microplate Reader and recorded every 9 seconds for 1 hour.

### Analysis of drug synergism

The Combination Index (CI) was calculated to determine whether the drugs interacted synergistically, additively, or antagonistically [14]. The CI is calculated by the following equation: CI = (D)_1_/(Dx)_1_+(D)_2_/(Dx)_2_+(D)_1_×(D)_2_/(Dx)_1×_(Dx)_2,_ in which (D)_1_ is the concentration of a drug necessary to achieve a particular effect in the combination; (Dx)_1_ is the concentration of the same drug that will produce the identical level of effect by itself; (D)_2_ is the concentration of the second drug that will produce a particular effect in the combination; and (Dx)_2_ is the concentration of the second drug, which will produce the same level of effect by itself. CI>1 indicates antagonism, CI <1indicates synergy, and CI = 1 indicates additivity [38]. Two independent experiments were performed to obtain the CI. In the first experiment, (D)_1_ = 10 µM, (Dx)_1_ = 25.84 µM, (D)_2_ = 20 nM, (Dx)_2_ = 92.79 nM, CI = 0.686; in the second experiment, (D)_1_ = 10 µM, (Dx)_1_ = 25.48 µM, (D)_2_ = 20 nM, (Dx)_2_ = 88.283 nM,CI = 0.572. The average CI = 0.63±0.08.
